# Effectiveness and Tolerability of Ectoin^®^ Mouth and Throat Spray Althaea Honey (ERS09) for Sore Throat due to Acute Pharyngitis and Dry Cough: A Multicentre, Actively Controlled, Open Label Study in Germany

**DOI:** 10.3390/jcm12185813

**Published:** 2023-09-07

**Authors:** Don Lorenzo Constantin Roventa, Ursula Pieper-Fürst, Cengizhan Acikel, Dunia Santos, Ulrike Sent, Ralph Mösges

**Affiliations:** 1 Institute of Medical Statistics and Bioinformatics (IMSB), Faculty of Medicine, University of Cologne, 50924 Cologne, Germany; roventa.constantin@gmail.com; 2 ClinCompetence Cologne GmbH, Theodor-Heuss-Ring 14, 50668 Cologne, Germany; ursula.pieper-fuerst@clincompetence.de (U.P.-F.); cengiz.acikel@clincompetence.de (C.A.); 3 Sanofi, 65926 Frankfurt, Germany; dunia.santos@sanofi.com (D.S.); ulrike.sent@sanofi.com (U.S.)

**Keywords:** acute pharyngitis, dry cough, ectoine, sore throat, throat spray, treatment device

## Abstract

Acute pharyngitis can cause sore throat. This multicentre, active-controlled, randomised, open-label, and parallel-group study, conducted according to the German Medical Devices Act, compared the effectiveness and tolerability of ERS09 mouth and throat spray with a well-established device for the treatment of sore throat caused by acute pharyngitis and dry cough. Patients were randomised 1:1 into ERS09/comparator groups (EMSER^®^ Sore Throat Spray) for 7 ± 2 days. Patients and investigators reported effectiveness (change in total symptom score [TSS]) and safety endpoints (incidence of adverse events [AEs]; adverse device effects [ADEs]). A total of 186 patients were included (ERS09: *n* = 92; comparator: *n* = 94). The baseline-adjusted mean TSS over 7 days was −90.14 and −74.91 in the ERS09 and comparator groups, respectively (*p* < 0.05). The majority of patients reached a 50% reduction in symptoms by day 6 (ERS09 = 78.85; comparator = 75.8%). Most patients reported a soothing effect within five minutes (ERS09 = 82%; comparator = 71%). Improvements in individual symptoms were similar with no significant differences between groups; more patients in the ERS09 group reported an improvement in pharyngeal redness/swelling. Three AEs unrelated to medication, one ADE following ERS09, and no serious AE/ADE were reported. ERS09 was as well tolerated and effective as the established device, showing greater improvement in the management of some symptoms and greater patient preference.

## 1. Introduction

Acute pharyngitis is a condition characterised by inflammation in the upper respiratory tract that causes sore throat and dysphagia [1]. Its aetiologies include a variety of pathogens, but viral infections (coronavirus, rhinovirus, adenovirus) are the most common, accounting for about 70–95% of cases [2]. Upper respiratory tract infections can cause acute cough, which is highly prevalent within a population at any given time [3]. Acute pharyngitis and dry cough can resolve without the need for treatment; however, the discomfort can be such that most patients seek treatment for their symptoms [4].

Sore throat and acute cough are some of the most common reasons for visits to general medicine physicians [2,5], and over-the-counter products, which treat symptoms, are widely used and recommended [1]. Laryngeal reflux is a predictive factor and may be associated with pharyngitis, so should be considered a differential diagnosis [6]. Cases of severe sore throat may require referral to a hospital or urgent care depending on the underlying cause and concurrent symptoms [7]. Acute sore throat and fever can be managed with oral analgesics, such as nonsteroidal anti-inflammatory drugs (NSAIDs) or paracetamol, but their use is limited by the risk of systemic adverse events and the low concentration of drug substance present in the affected area [1]. The current guideline from the German Society for General Medicine and Family Medicine (DEGAM) for the treatment of sore throat recommends medicinal and non-medicinal lozenges, local analgesics, and/or NSAIDs for relieving symptoms. Furthermore, a short-term symptomatic therapy with ibuprofen or naproxen can be offered for acute sore throat [8].

To limit the side effects of these medications, an alternative treatment is of considerable interest. The oropharyngeal cavity is easily accessible, so pharmaceutical agents can be directly administered to the target site. One preferable, convenient, and highly targeted remedy for sore throat is the use of a throat spray. The soft mist dispersed from the throat spray easily reaches the inflamed tissue in the back of the throat to provide targeted and fast relief [9].

ERS09 (Bitop AG, device brand name Ectoin^®^ Mouth & Throat Spray Althaea Honey) is a medical device containing a combination of ectoine, *Althaea officinalis*, and honey that may provide a multi-modal symptomatic treatment for sore throat due to acute pharyngitis and dry cough. The ectoine molecular compound is a cyclic amino acid derivative with osmoregulatory function [10].

The aim of this study (NCT04203810), conducted according to the German Medical Devices Act, was to investigate the clinical effectiveness and tolerability of ERS09 compared with a well-established throat spray (EMSER^®^ Hals-und Rachenspray) for the symptomatic treatment of sore throat due to acute pharyngitis and dry cough in a representative sample of German adolescents and adults.

## 2. Materials and Methods

### 2.1. Study Design

This was a multicentre, active-controlled, randomised, open-label, and parallel-group study (NCT04203810) conducted between January and December 2020 in Germany. This head-to-head comparative study of medical devices was performed in compliance with the Good Clinical Practice guidelines and the principles of the Declaration of Helsinki. Informed consent was obtained from all patients prior to enrolment. The study was designed and undertaken according to the local German Medical Devices Act (§ 23b MPG, valid until 25 May 2021) [11], which aims to make new medical devices rapidly available for patients and users while guaranteeing safety, suitability, and performance. This act does not require the use of a placebo-controlled group or blinding for clinical investigation (including evaluation of adverse events) if certain criteria are met. Paragraph 23b of the German Medical Devices Act states the exceptions to the provisions governing clinical investigations; exceptions are valid when the clinical investigation is conducted using devices which are authorised in accordance to bear the CE marking (unless the aim of the investigation is to use the device for a different intended purpose, or additional invasive or other stressful examinations are to be carried out). As the certified devices used fell into these criteria, application within their intended use was mandatory, as was precluding a placebo group and blinding.

The study was originally planned to be conducted between October 2019 and June 2020, but was delayed until January 2020 due to awaiting ethical approval and extended until December 2020 due to decreased recruitment during the COVID-19 pandemic.

Patients attended 3 visits: visit 1 (enrolment), visit 2 (day 3 ± 1 of treatment), and visit 3 (final exam day 7 ± 2 of treatment). Patients reported responses using a questionnaire in a patient diary, and subjective data were assessed via patient questionnaire at visit 1 and visit 3. Objective data from the examination of the patients were also reported by the investigator (physician) via an investigator questionnaire (Table S1). Discontinuation criteria included: any adverse event (AE), severe adverse event (SAE), adverse device effect (ADE), or serious adverse device effect (SADE) impairing further participation in the study; development of non-inclusion criteria after the study enrolment by the patient; or the patient was not able to adhere to the study protocol or desired to be withdrawn from study participation.

### 2.2. Participants

Patients were included if they were aged 12 years or older with sore throat due to acute pharyngitis and dry cough, with onset of symptoms no more than 72 h prior to visit 1 and a sore throat pain intensity score ≥ 40 mm, as measured on a 100 mm visual analogue scale (VAS) (0 mm representing ‘no pain’ to 100 mm representing ‘worst possible pain’).

Patients were excluded if they were known to be hypersensitive to ectoine, *Althaea officinalis*, honey, and/or other ingredients in ERS09 or the comparator; were fructose intolerant or had glucose–galactose malabsorption; if they had suspected bacterial pharyngitis; symptoms lasting longer than 72 h; used any pain or cough medication within 24 h preceding enrolment; had oral lesions or oral surgical procedures within 1 month prior to enrolment; were pregnant or breastfeeding; or based on the Investigator’s discretion (e.g., patients with a history of drug abuse or a psychiatric disorder as well as patients unwilling to give informed consent or abide by the requirements of the protocol).

### 2.3. Study Treatments

Patients were treated either with ERS09 (4 puffs as needed several times a day up to 10 applications daily) or comparator, EMSER^®^ Sore Throat Spray (1–3 puffs several times daily) for 7 ± 2 days. ERS09 was composed of ectoine, *Althaea officinalis*, honey, water, maltodextrin, silicon dioxide, and potassium sorbate. The comparator (100 mL) was composed of 1.175 g natural Emser salt and purified water. Paracetamol 500 mg (up to 4 times daily) and/or Xylometazoline 0.1% (1 puff per nostril up to 3 times daily) were permitted for use as rescue medications, which is in line with current German guidelines for the symptomatic treatment of sore throat and rhinosinusitis [8,12].

### 2.4. Study Endpoints and Analysis

Endpoints were reported by patients or investigators (physicians) via a questionnaire at each visit (Table S1). Study endpoints to assess the clinical effectiveness of ERS09 included the baseline-adjusted mean change in Total Symptom Score (TSS) from day 0 to day 7 (combining sore throat intensity, pain on swallowing, and dry cough severity as reported in the patient diary), and the baseline-adjusted daily improvement in TSS for individual symptoms. TSS was self-reported via a patient diary and individual symptoms were reported using a Visual Analogue Scale (VAS) 100 mm in length (0 mm representing ‘no pain’ to 100 mm representing ‘worst possible pain’). Pharyngeal swelling and redness were reported by the investigator at each visit (assessment scale: 0 = none, 1 = mild, 2 = moderate, 3 = severe). Times relating to a 50% reduction in symptoms, as shown by TSS score and effect on sleep impairment (0 = no impairment, 1 = slight impairment, 2 = moderate impairment, 3 = severe impairment), were also assessed.

Further effectiveness exploratory endpoints included an evaluation of the effect duration (assessed by the time between the first and second administration as reported in the diary), the onset of the soothing effect of the throat spray, the likelihood of reuse of the throat spray by the patient, the likelihood of the patient to recommend the spray to friends and family, the patient and physician assessment of overall effectiveness, the taste of the throat spray using a scale from 1 to 100 (0 = very unpleasant taste; 100 = very pleasant taste), and the intake of the rescue medication which was assessed for the use of paracetamol (up to 4 pills daily or 2000 mg) and the use of the xylometazoline 0.1% nasal spray (up to 3 puffs daily).

Safety endpoints included the occurrence of any AEs, ADEs, SAEs, and SADEs according to the Medical Dictionary for Regulatory Activities (MedDRA) code classifications. Assessments of the tolerability of the therapy were undertaken by investigators and patients.

### 2.5. Randomisation

Patients were randomised in a 1:1 ratio into each of the study groups based on a computer-generated block randomisation list. Patients were assigned to a treatment in the individual treatment centres in ascending order to the next randomisation number (open label).

### 2.6. Statistical Methods

The intention-to-treat (ITT) population included all randomised patients who had at least one day of TSS data. The modified ITT (mITT) population contained all randomised patients with TSS data for the entire observation period (day 0 to day 5 at least). The safety population included all randomised patients who had been exposed to treatment at least once. The mITT population was considered for analysis of TSS endpoints, evaluation of the effect duration, and intake of the rescue medication, and the ITT population was considered for analysis of other study endpoints.

Study endpoints were presented by descriptive statistics; their changes from day 0 to days 7 ± 2 were displayed. Descriptive and exploratory analyses were performed using the International Business Machine’s Statistical Package for the Social Sciences (IBM SPSS) Statistics for Windows, version 26.0.0.1 (Armonk, NY, USA: IBM Corp.). Descriptive statistics for continuous variables were represented by count, mean, standard deviation, minimum, maximum, and quartiles. Categorical variables were represented by frequency and valid percentages. Overall TSS and individual symptom scores were analysed using the global linear model (GLM) and analysis of variance (ANOVA). For global linear modelling, TSSs at the last visit were used as the dependent variable and the effect of treatment was assessed via adjustment of baseline scores. Normality analyses were performed using the One-Sample Kolmogorov–Smirnov test, then continuous variables between groups were compared using the Mann–Whitney U test. For all exploratory statistical comparisons, a two-tailed *p*-value ≤ 0.05 was considered statistically significant.

Due to the new formulation of ERS09 and the administration of the comparator as a throat spray, a pronounced reduction in symptoms and little influence of the comparator was expected. To calculate the necessary sample size, it was assumed that both treatment groups would present identical mean baseline values in the TSS of 180 mm (3 × VAS, each of 60 mm) during enrolment. A linear decrease in symptoms (40% decrease for ERS09 [mean AUCERS09 = 144]; a 20% decrease for comparator [AUCcomparator = 144] after 8 days of treatment), a standard deviation σ = 72 units, a two-sided α = 0.05, and a power of 90% were assumed to calculate a sample size of 86 patients per treatment group. With an expected dropout rate of 20%, 216 patients in total for both groups needed to be randomised to result in 172 patients to complete the study and be eligible for analysis.

## 3. Results

**Figure 1:**
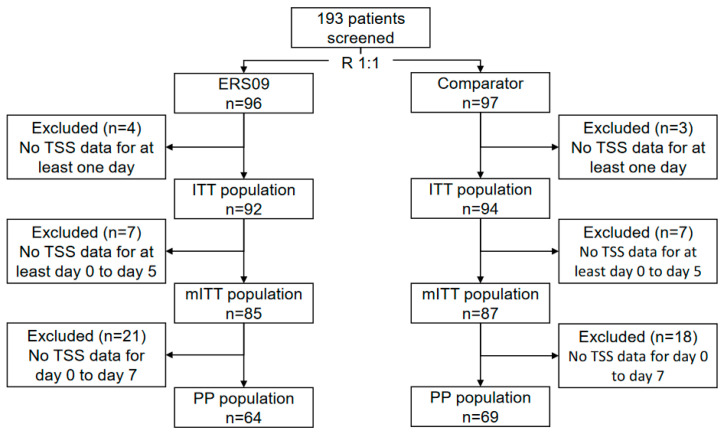
Study flowchart. ITT, intention-to-treat; mITT, modified intention-to-treat; PP, per protocol; TSS, total symptom score.

The ERS09 treatment group included 96 patients (49.7%), and the comparator treatment group included 97 patients (50.3%) (Figure 1). The two treatment groups were considered balanced in gender (ERS09 67.4% female; comparator 68.1% female) and age (ERS09 mean age 41.89 years; comparator mean age 42.82 years). A total of 186 patients completed the treatment with ERS09 (*n* = 92) or comparator (*n* = 94).

### 3.1. Reduction in TSS

**Figure 2:**
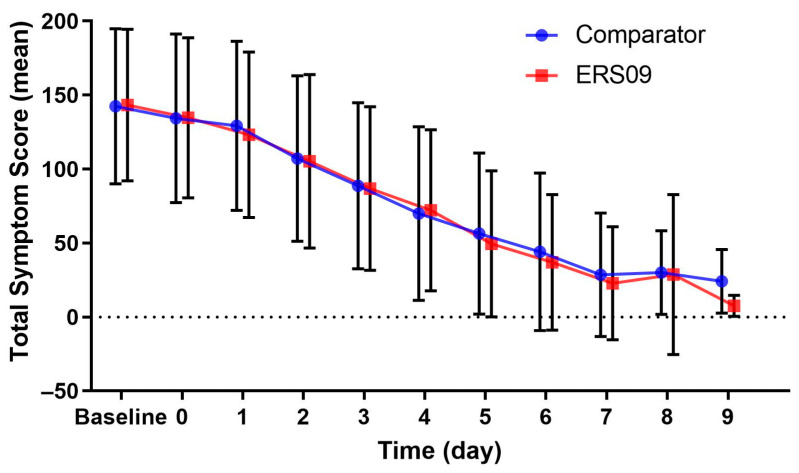
Mean total symptom score from baseline over 9 days of treatment for both treatment groups. Bars show standard deviation.

The mean TSS at baseline was similar in each treatment group. In the ERS09 group, the mean TSS decreased from 143.28 at baseline to 49.48 on day 5, 22.80 on day 7, and 7.50 on day 9. In the comparator group, the mean TSS decreased from 142.49 at baseline to 56.34 on day 5, 28.45 on day 7, and 24.14 on day 9 (Figure 2).

**Figure 3:**
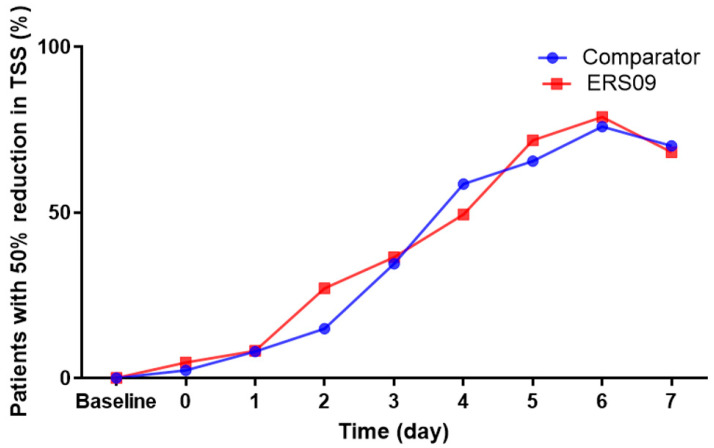
Proportion of patients with 50% reduction in TSS in each treatment group. TSS, total symptom score.

The majority of patients reached the 50% reduction threshold by day 6 (78.8% in the ERS09 group compared to 75.8% in the comparator group) (Figure 3). In the subgroup of patients who achieved this threshold by day 3, the baseline-adjusted mean TSS over 7 days was −90.14 and −74.91 in the ERS09 and comparator groups, respectively. The value is significantly lower in the ERS09 group (*p* < 0.05), demonstrating the superiority of the device among this subgroup of patients.

### 3.2. Individual Symptoms

**Figure 4:**
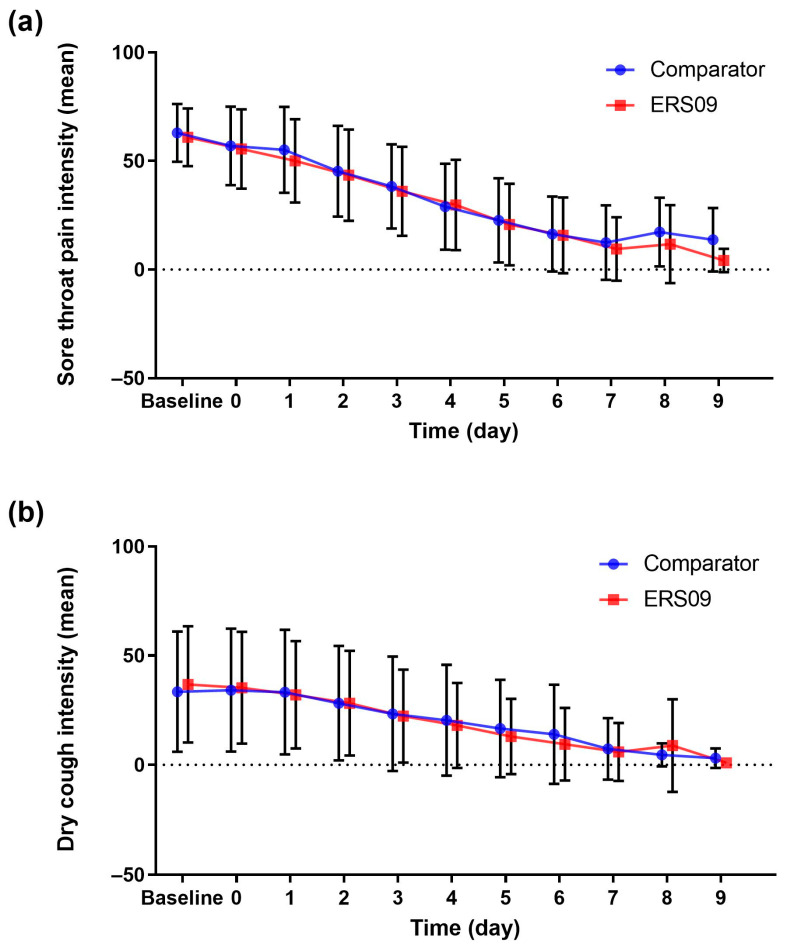
Mean intensity of (**a**) sore throat pain and (**b**) dry cough in both treatment groups. Bars show standard deviation.

The mean sore throat pain intensity over 7 treatment days was 32.51 in the ERS09 group and 35.22 in the comparator group, with no significant difference reported at baseline across groups (*p* = 0.330). In the ERS09 group, the mean sore throat pain intensity decreased from 60.94 at baseline to 20.69 on day 5, 9.46 on day 7, and 4.17 on day 9. There was no statistically significant difference in the reduction in sore throat pain between study groups (*p* = 0.843) (Figure 4).

In the ERS09 group, mean severity of dry cough decreased from 36.82 at baseline to 13.08 on day 5, 5.89 on day 7, and 1.00 on day 9 (Figure 4). There was no statistically significant difference in the reduction in dry cough severity between study groups (*p* = 0.978).

In the ERS09 group, the mean pain on swallowing decreased from 45.52 at baseline to 15.71 on day 5, 7.43 on day 7, and 2.33 on day 9. The baseline-adjusted pain/difficulties on swallowing averaged over 7 treatment days was −29.45 in the ERS09 group and −32.53 in the comparator group. There was no statistically significant difference in the reduction in pain on swallowing between study groups (*p* = 0.815).

The proportion of patients with an improvement in pharyngeal swelling and redness increased at each visit, and at the end of the study period was 5.9% and 5.8%, respectively, higher in the ERS09 group than in the comparator group (Figure S1). A statistically significant difference across study groups was not detected (*p* = 0.402).

The proportion of patients who reported moderate and severe sleep impairments decreased continuously from 36.2% on day 0 to 12.0% on day 4 in the ERS09 group. On day 5, the value for this proportion of patients was 7.2% and increased to 9.1% on day 8. On day 9, there were no moderate or severe sleep impairment reported. A similar improvement was seen in the comparator group with no statistically significant difference between groups (*p* = 0.801).

### 3.3. Further Investigations

The mean time between doses was 3.34 h in the ERS09 group and 4.08 h in the comparator group, up to a maximum time span of 13.67 h in the ERS09 group and 13.50 h in the comparator group. The difference was significant between the groups and was higher in the comparator group (*p* < 0.05). In the ERS09 group, significantly more patients reported that they would reuse the throat spray should they experience pharyngitis symptoms in the future; 88.6% of patients versus 76.9% of patients from the comparator group (*p* = 0.018). When asked to evaluate the treatment, the majority of patients in the ERS09 group (89.8%) reported that they would recommend the throat spray to friends and family, but slightly fewer patients in the comparator group answered the same (81.3%). The difference was significant between groups (*p* = 0.023).

Overall effectiveness was assessed by the patient and the physician at visit 3. In the ERS09 and comparator groups, 87.8% and 82.6% of physicians, respectively, rated the spray as very good or good (*p* = 0.103). The patients’ assessment of effectiveness showed similar results, with 79.5 % and 73.6% of patients rating the spray as very good or good in the ERS09 and comparator groups (*p* = 0.066). Most patients reported a soothing effect within the first 5 min after application; 82.2% of ERS09 patients and 71.1% of comparator patients, respectively (*p* = 0.144). Patients assessed the taste of throat spray; the median was 63.50 and 63.00 in the ERS09 group and in the comparator group, respectively (*p* = 0.821). The difference between groups was not statistically significant (*p* = 0.144).

The majority of patients (~60% on day 0 and day 1) did not use paracetamol as rescue medication in either group, whereas 51.4% of patients in the comparator group and 42.4% of patients in the ERS09 group never used xylometazoline during the study. The difference was not statistically significant (*p* = 0.293) (Table 1).

### 3.4. Safety

Three AEs not related to the medical devices occurred: one case of bronchitis (ERS09 group) and two cases of bacterial pharyngitis (comparator group). The patients required treatment with antibiotics and so discontinued the study. One ADE occurred in this study: one patient in the ERS09 group experienced mild swelling of the pharyngeal mucosa following regular application of the throat spray. The patient stopped the application of the throat spray and discontinued the study after visit 2. No serious AE or ADE occurred in this study.

The results of the study have been summarised in Supplementary S1.

## 4. Discussion

This local German study, conducted according to the German Medical Devices Act, aimed to evaluate the effectiveness and tolerability of the multi-modal medical device ERS09, compared with the ‘gold-standard’ comparator medical device, for the symptomatic treatment of sore throat due to acute pharyngitis and dry cough. ERS09 mouth and throat spray was demonstrated to be at least as effective and well tolerated as the comparator. ERS09 treatment improved acute pharyngitis symptoms throughout the study and was well perceived by patients, a high proportion of whom would reuse this device if they developed symptoms in the future and would recommend this therapy to others. The ERS09 group had a higher proportion of patients with improved mild pharyngeal swelling or redness from visit 1 to visit 3 in contrast to the comparator group, indicating more effective resolution of these symptoms with ERS09. The comparator used in this study is considered the ‘gold-standard’ in the German market as it is effective and well tolerated in the treatment of acute pharyngitis [13]. In the current study, effectiveness endpoints, such as the TSS score, improved in a similar manner over the course of treatment with both devices, demonstrating equal effectiveness of the new treatment device ERS09. However, in the field of over-the-counter medications, patients’ preference and adherence to the therapy play an important role for the clinical relevance of the treatment considered in addition to its effectiveness and tolerability [14]. The current study showed significantly greater patient preference for the new device, in relation to the probability of reuse and recommendation of the throat spray as well as a higher rating for taste, suggesting that ERS09 could be a viable alternative treatment for sore throat relief.

Symptom burden of acute pharyngitis and sore throat largely impact the quality of life (QoL) of patients and place a great burden on healthcare systems. It is estimated that about 30% of a population have a sore throat in any one year [15], and 1–2% of all consultations in primary care are due to patients seeking treatment advice about a sore throat [16]. In cases where pharyngitis has chronic status based on the presence of lingual tonsillitis, where surgical intervention may be considered, the use of robotic support may be helpful [17]. Acute cough can affect an even greater proportion of the population, up to 64% at any given time [3], and can account for up to 10 outpatient medical services per 1000 visits each year in the United States alone [18]. Treatment of symptoms is therefore recommended to relieve discomfort, prevent disruption to daily activities, and improve quality of life. During this study, it was shown that ERS09 treatment improved sleep on a par with the comparator, suggesting that QoL outcomes can be effectively improved with this treatment.

The local and targeted treatment of symptoms via a throat spray offers an alternative and a convenient treatment without the need for systemic treatment with NSAIDs. The current guidelines for the management of sore throat state that there is little evidence to support the use of traditional or natural therapies. However, previous studies have looked at the effect of ectoine; the primary mode of action of ectoine is non-pharmacological and is exerted through the interaction of water with the solute, providing a stabilising effect of the ectoine–hydro complex on the epithelial tissue treated [19], and pre-clinical models have shown that this reduces the inflammatory process at the membrane level [20]. Previous studies have also shown that ectoine, when administered by oral or nasal spray, effectively reduces inflammation, symptoms of acute pharyngitis, rhinosinusitis, and allergic rhinitis [13,21], and is well tolerated [22,23,24]. Moreover, two clinical trials have demonstrated the effectiveness of this multi-modal treatment as a throat spray in comparison to saline lozenges, and as lozenges in comparison to hyaluronic acid lozenges and saline gargling. Both studies demonstrated the superiority of ectoine-based products [13,25]. This current study supports the use of ERS09 as an effective and well-tolerated natural therapeutic device for sore throat due to acute pharyngitis and dry cough.

Limitations of this study include the fact that, firstly, the comparison of the effectiveness of the two devices used in this study was exploratory only; a controlled study could not be conducted due to the different composition of the active ingredients in ERS09 and the comparator. However, according to the local Medical Devices Act, blinding and placebo were precluded from the study design. Secondly, the TSS analysis included patients who had taken rescue medication, which may have masked their symptoms. However, there was no statistically significant difference in the use of paracetamol or Xylometazoline between groups, so this effect may be considered balanced between groups.

In conclusion, both throat sprays were shown to be well tolerated and effective for the treatment of acute pharyngitis. There was greater improvement shown in the resolution of some symptoms with the new multi-modal treatment device compared to the well-established device, patients preferred using the new device and were more likely to recommend this therapy. Findings from this study suggest that ERS09 may be used as an alternative over-the-counter treatment for the relief of sore throat.

## Figures and Tables

**Table 1:** Intake of rescue medication in both treatment groups.

	ERS09 (*n* = 85)	Comparator (*n* = 87)
Paracetamol use, n (%)		
Day 0	31 (36.5)	33 (37.9)
Day 1	32 (37.6)	34 (39.1)
Day 2	27 (31.8)	34 (39.1)
Day 3	20 (23.5)	30 (34.5)
Day 4	17 (20.0)	22 (25.3)
Day 5	13 (15.3)	19 (21.8)
Day 6	8 (9.9)	16 (19.0)
Day 7	4 (6.1)	7 (10.0)
Day 8	0 (0.0)	3 (5.0)
Day 9	0 (0.0)	0 (0.0)
Xylometazoline nasal spray use, n (%)		
Day 0	36 (42.4)	35 (40.2)
Day 1	43 (50.6)	35 (40.2)
Day 2	34 (40.0)	38 (43.7)
Day 3	35 (41.2)	36 (41.4)
Day 4	29 (34.1)	30 (34.5)
Day 5	28 (32.9)	27 (31.0)
Day 6	19 (23.5)	21 (25.0)
Day 7	11 (16.7)	16 (22.9)
Day 8	0 (0.0)	9 (45.0)
Day 9	0 (0.0)	2 (28.6)

## Data Availability

Data are available from the corresponding author upon request.

## Supplementary Materials

The following supporting information can be downloaded at: https://www.mdpi.com/article/10.3390/jcm12185813/s1. Table S1: Patient and investigator questionnaires and patient diary; Figure S1: Percentage of patients with different intensity of (a) pharyngeal swelling (b) pharyngeal redness; Figure S2: Infographic summary of the study; Supplementary S1: Plain language summary of the study.
