# Supplementary material for: Effectiveness and Tolerability of Ectoin® Mouth and Throat Spray Althaea Honey (ERS09) for Sore Throat due to Acute Pharyngitis and Dry Cough: A Multicentre, Actively Controlled, Open Label Study in Germany
Source: J Clin Med. 2023 Sep 7;12(18):5813. doi: 10.3390/jcm12185813 (PMC10531782; doi:10.3390/jcm12185813)
Supplement: Supplementary file 1 [file jcm-12-05813-s001.zip › jcm-2530947-supplementary.pdf]

Effectiveness and Tolerability of Ectoin® Mouth and Throat Spray Althaea Honey (ERS09) for Sore Throat due to Acute Pharyngitis and Dry Cough: A Multicentre, Actively Controlled, Open Label Study in Germany

Don Lorenzo Constantin Roventa [1], Ursula Pieper-Fürst [2], Cengizhan Acikel [2], Dunia Santos [3], Ulrike Sent [3] and Ralph Mösges [1,2,*]

[1] Institute of Medical Statistics and Bioinformatics (IMSB), Faculty of Medicine, University of Cologne,
50924 Cologne, Germany; roventa.constantin@gmail.com
[2] ClinCompetence Cologne GmbH, Theodor-Heuss-Ring 14, 50668 Cologne, Germany; ursula.pieper-fuerst@clincompetence.de (U.P.-F.); cengiz.acikel@clincompetence.de (C.A.)
[3] Sanofi, 65926 Frankfurt, Germany; dunia.santos@sanofi.com (D.S.); ulrike.sent@sanofi.com (U.S.)
* Correspondence: ralph@moesges.de

Table S1. Patient and investigator questionnaires and patient diary

Patient diary	
The use of rescue medication will be assessed daily using the following score	0 = no intake of paracetamol tablets<br>1 = intake of up to 2 tablets of paracetamol daily<br>2 = intake of up to 4 tablets of paracetamol daily<br>3 = intake of more than 4 tablets of paracetamol daily<br><br>0 = no administration of xylometazoline<br>1 = administration of 1 puff per nostril of xylometazoline daily<br>2 = administration of 2 puffs per nostril of xylometazoline daily<br>3 = administration of 3 puffs per nostril of xylometazoline daily
In addition, patients will be asked if their sleep was impaired.<br><br>The sleep impairment due to sore throat and dry cough will be assessed using the following score	0 = no impairment<br>1 = slight impairment<br>2 = moderate impairment<br>3 = severe impairment
Only at V1, patients, will be asked to evaluate the effect duration by assessing the time span between the first and second spray administration (4, 6, 8 hours or more)	
Investigator questionnaire	
At all visits, an oropharyngeal examination will be performed, assessing the redness of pharynx, swelling of pharynx, swelling of lymph nodes using the following assessment scales:	0 = none<br>1 = mild<br>2 = moderate<br>3 = severe
At all visits, investigators will assess the	0 = very poor

patients' general condition, using the following scale	1 = poor<br>2 = moderate<br>3 = good
In addition, fever will be measured at each visit and documented using the following scale	Normal temperature: 36-37°C<br>Raised temperature: 37-38°C<br>Fever: ≥ 38°C<br>High fever: ≥ 39°C
At V3, investigators are asked to give a general assessment of the therapy<br><br>Investigators are asked to evaluate the overall effectiveness.<br><br>Investigators are asked to evaluate the overall tolerability of the therapy	0 = Non-satisfactory<br>1 = Satisfactory<br>2 = Good<br>3 = Very good<br><br>0 = Non-satisfactory<br>1 = Satisfactory<br>2 = Good<br>3 = Very good
Patient Questionnaire	
1. Patients are asked at visit 1 to assess the soothing effect of the throat spray immediately after the first application in the patient questionnaire. If immediately after application no soothing effect was perceived, the patient is asked after 1, 2, 5, 10 minutes to assess the timepoint of onset of soothing.	
2. Patients are asked to evaluate the overall effectiveness of the therapy	0 = Non-satisfactory<br>1 = Satisfactory<br>2 = Good<br>3 = Very good
3. Patients are asked to evaluate the overall tolerability of the therapy	0 = Non-satisfactory<br>1 = Satisfactory<br>2 = Good

	3 = Very good
4. Patients are asked to answer the question: “Would you reuse the throat spray during your next cold episode?”	0 = I would not reuse this therapy in any case.<br>1 = I would probably not reuse this therapy.<br>2 = I would probably reuse this therapy.<br>3 = I would definitely reuse this therapy.
5. Patients are asked to answer the question: “Would you recommend the throat spray to your friends and family?”	0 = I would not recommend this therapy in any case.<br>1 = I would probably not recommend this therapy.<br>2 = I would probably recommend this therapy.<br>3 = I would definitely recommend this therapy.
6. Patients are asked to assess the taste of the throat spray by using Visual Analogue Scale (VAS), 100 mm in length, and anchored by two verbal descriptors, one for each extreme symptom	

V, visit

Figure S1. Percentage of patients with different intensity of a) pharyngeal swelling b) pharyngeal redness

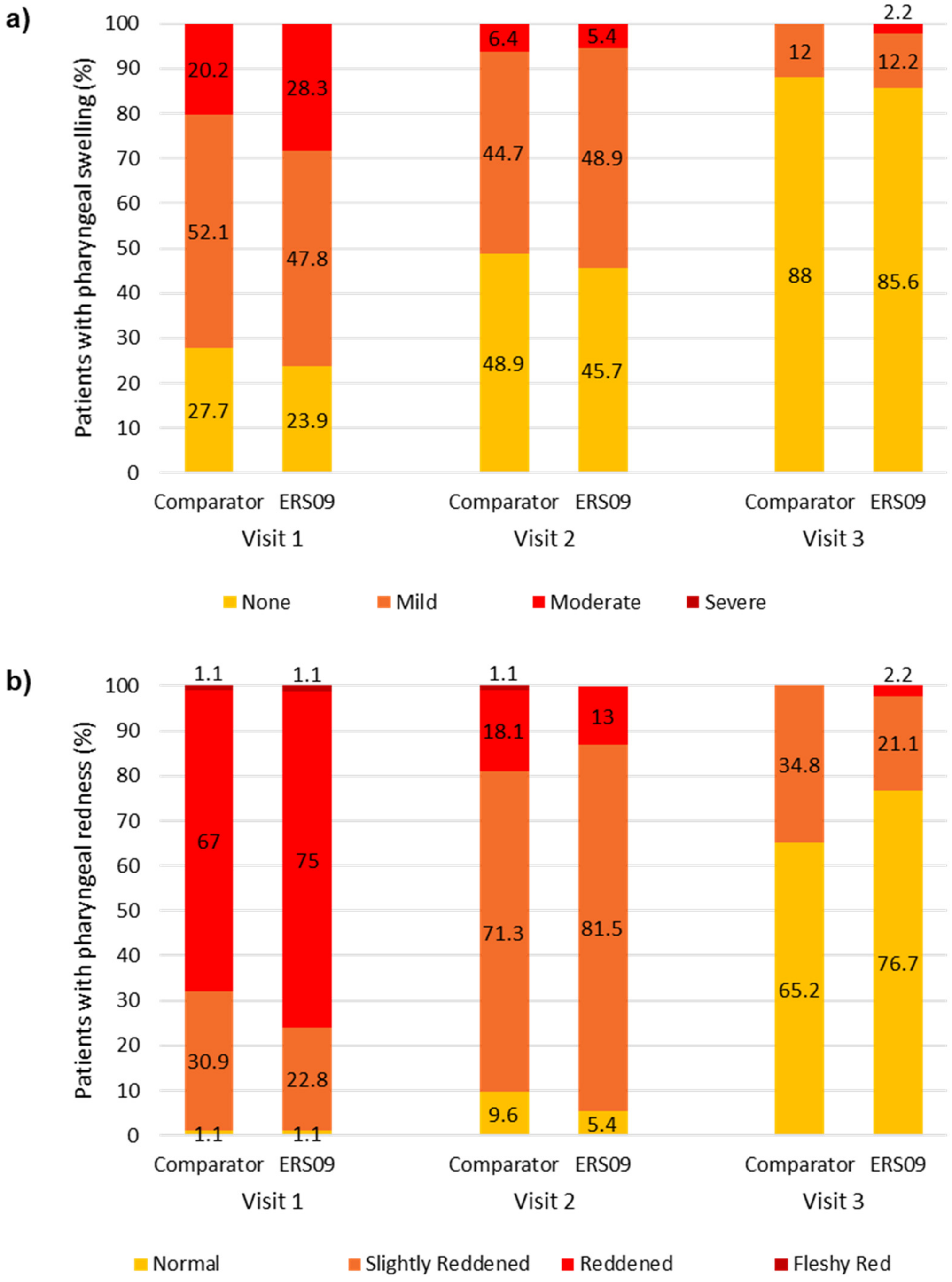

Figure S2. Infographic summary of the study

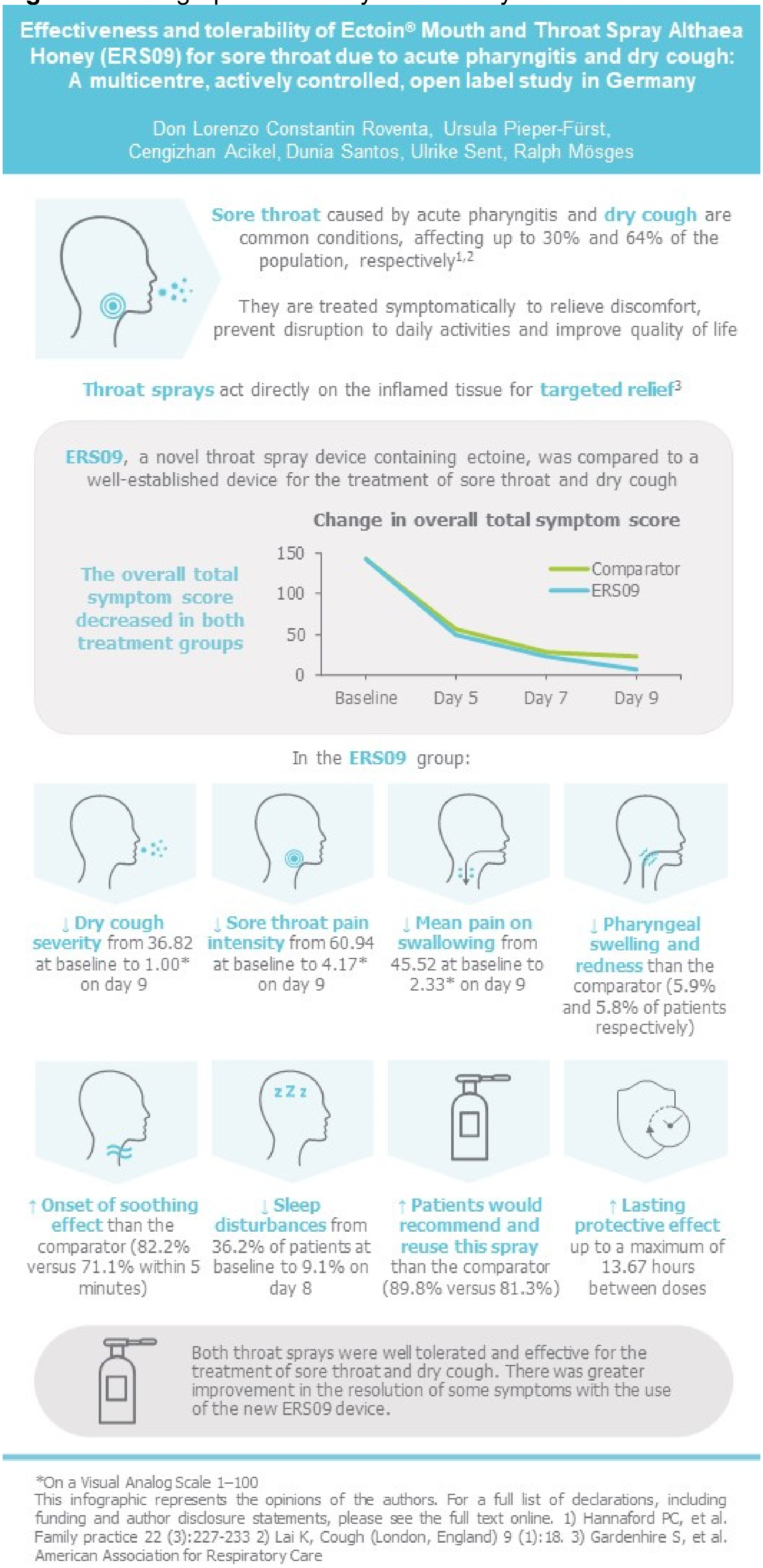

1. Hannaford PC, Simpson JA, Bisset AF, Davis A, McKerrow W, Mills R. The prevalence of ear, nose and throat problems in the community: results from a national cross-sectional postal survey in Scotland. *Fam Pract* **2005**, 22, 227-233.
2. Lai K, Pan J, Chen R, Liu B, Luo W, Zhong N. Epidemiology of cough in relation to China. *Cough* **2013,** 9, 18
3. Gardenhire, S.; Burnett, D.; Strickland, S.T.M. *A Guide To Aerosol Delivery Devices for Respiratory Therapists*; American Association for Respiratory Care: Irving, TX, USA, 2017.

Supplementary S1: Plain language summary of the study

Acute pharyngitis, the inflammation of the pharynx, and dry cough are common conditions usually caused by viruses and presenting symptoms of sore throat (e.g. pain on swallowing, swelling/redness of the throat). Patients can self-manage symptoms with over-the-counter products, such as oral analgesics. However, side effects affecting the whole body limit the use of these medicines, thus the development of local treatments is of interest. This study compared a new throat spray with a well-established device for the treatment of sore throat caused by acute pharyngitis and dry cough. The new throat spray contains ectoine to relieve soreness. Patients were assigned to use either the new or the well-established device for 7 days. During the study, patients and investigators scored the improvement in selected symptoms (e.g. pain on swallowing) and treatment satisfaction. The total score of all symptoms decreased by half in both treatment groups, including 80% of those using the new device. Each symptom improved similarly in both groups, but patients who used the new device reported a greater improvement in swelling and redness of the back of the throat. Overall, patients using the new device were more satisfied with their treatment. One mild side effect following application of the new spray occurred. No serious side effects or side effect caused by the device occurred in this study. These findings support the use of the new device for the symptomatic treatment of sore throat in patients with acute pharyngitis and dry cough.
